# Robotic technology (ROBERT^®^) to enhance muscle strength in the hip flexor muscles following spinal cord injury: a feasibility study

**DOI:** 10.1038/s41394-024-00630-9

**Published:** 2024-04-10

**Authors:** S. L. Sørensen, I. Poulsen, L. A. Harvey, F. Biering-Sørensen, J. F. Nielsen

**Affiliations:** 1 Department of Neurology, Spinal Cord Injury Centre of Western Denmark, Regional Hospital Viborg, Viborg, Denmark; 2 Department of Clinical Medicine, Aarhus University, Aarhus, Denmark; 3 Department of Clinical Research, Copenhagen University Hospital, Amager and Hvidovre, Denmark; 4 Department of People and Technology, Roskilde University, Roskilde, Denmark; 5 Research Unit Nursing and Health Care, Health, Aarhus University, Aarhus, Denmark; 6 John Walsh Centre for Rehabilitation Research, Kolling Institute, Faculty of Medicine and Health, University of Sydney, St. Leonards, NSW Australia; 7 Department for Brain- and Spinal Cord Injuries, Rigshospitalet, Copenhagen, Denmark; 8 Department for Clinical Medicine, University of Copenhagen, Copenhagen, Denmark; 9 Hammel Neurorehabilitation Centre and University Clinic, Aarhus University, Aarhus, Denmark

**Keywords:** Rehabilitation, Preclinical research

## Abstract

**Study design:**

Feasibility study.

**Objective:**

To determine the feasibility of conducting a large trial designed to determine whether the ROBERT^®^ can be used to increase the strength of the hip flexor muscles after spinal cord injury (SCI). The ROBERT^®^ is a robotic device that provides assisted active movement while supporting the weight of the leg. Focus was on recruitment capability, suitability, and acceptability of the intervention and outcome measure.

**Setting:**

Specialised SCI centre in Denmark.

**Methods:**

All first-time admitted patients were screened to assess participant recruitment capability. Four people with SCI < 3 months tested a protocol consisting of 60 repetitions of hip flexion in supine conducted with the assistance of the ROBERT^®^ three times a week for 4 weeks. Feasibility was assessed based on adherence to the protocol and completion rate and from the participants’ perspectives. Maximal voluntary contraction (MVC) was accessed at baseline and four weeks.

**Results:**

The recruitment rate was 8% (7 months). The four participants completed 44 out of 48 sessions (92%). No adverse events occurred. One physiotherapist was required to set-up and supervise each session. The active exercise time varied from 7.5 to 17 min. The participants found the ROBERT^®^ a good supplement to their usual rehabilitation. We were able to measure MVC in even very weak hip flexor muscles with a dynamometer MicroFET2 fixed to a frame.

**Conclusion:**

The ROBERT^®^ was feasible and acceptable. The participants perceived it as a supplement, not a replacement to usual physiotherapy. However, recruitment to the study was slow.

**Trial registration:**

ClinicalTrials.gov NCT05558254. Registered 28th September 2022.

## Introduction

There is a lack of evidence of how to increase voluntary muscle strength following spinal cord injury (SCI) in muscles with grade 3 strength or less (according to a manual muscle test; MMT) [1, 2]. Systematic reviews provide clear guidance on appropriate training interventions to increase the strength of neurologically intact muscles (eg., the upper limbs of people with paraplegia) but little guidance on training interventions for muscles with MMT grade 3 or less strength [1, 3–5]. It is speculated that different approaches to increase muscle strength are called for in neurologically weak muscles following SCI particularly in the weak (ie., grade 3 and less) [6, 7]. Muscles grade 3–5 appear to respond to the principles of progressive muscle training [6, 8] consisting of a small number of repetitions with high resistance. However, people with grades 1–2 and to some extent grade 3 are not capable of performing progressive muscle training. It has been suggested that instead, these very weak muscles may best respond to repetitive contractions. Yet, a multicentre randomised controlled trial (RCT) (*n* = 120) in people with subacute SCI found that 10,000 contractions of very weak muscles (grade 1–2) over 8 weeks had only a small or no effect on voluntary strength [7]. The effect may have been greater if the therapists had provided assisted active movement. This would have provided the opportunity for muscles to fire throughout range of motion (ROM). We were interested in this possibility, but it is very time-consuming and physical demanding for therapists to provide many repetitions of assisted active movement. This barrier can be overcome with robotics which provide graded assistance that can be gradually reduced as a patient gets stronger and ultimately replaced with graded resistance [9].

The ROBERT^®^ (Life science Robotics, Aalborg, Denmark) [10] is an example of a robotic device that provides assisted active movement. The movement of the ROBERT^®^ can be programmed to follow a recorded movement pattern. For example, the therapist can assist the person with grade 1 in the hip flexor muscles to perform full-range hip flexion. The ROBERT^®^ can then copy this movement without the need for the therapist. The ROBERT^®^ supports the weight of the limb enabling those with even grade 2 or less strength to move against gravity. As the patient regains strength, it can add resistance.

As there is no research literature exploring the use of the ROBERT^®^ as an intervention to increase strength in very weak muscles, we conducted a feasibility study. We plan to follow this up with a pilot study and then ultimately a future definitive trial. A feasibility study is understood as an iterative, formative, and adaptive study to assess the research and intervention process—and to answer the question “*can the study be done*?” [11, 12]. Specifically, we aimed to look at the ROBERT^®^ for providing assisted active hip flexion. We looked at hip flexion because it is particularly difficult for therapists to provide high repetitions of assisted active hip flexion. We focused on the subacute phase because of pragmatic reasons and because patients with SCI are probably most receptive to strength training intervention at this time. We did not plan to use the results of this feasibility study to guide sample size estimations.

## Objective

The objective of this study was to determine the feasibility of conducting a large trial designed to determine whether the ROBERT^®^ can be used to increase strength in the hip flexor muscles after SCI. Specifically, we focused on recruitment capability, suitability, and acceptability of the intervention and outcome measures.

## Methods

**Fig. 1:**
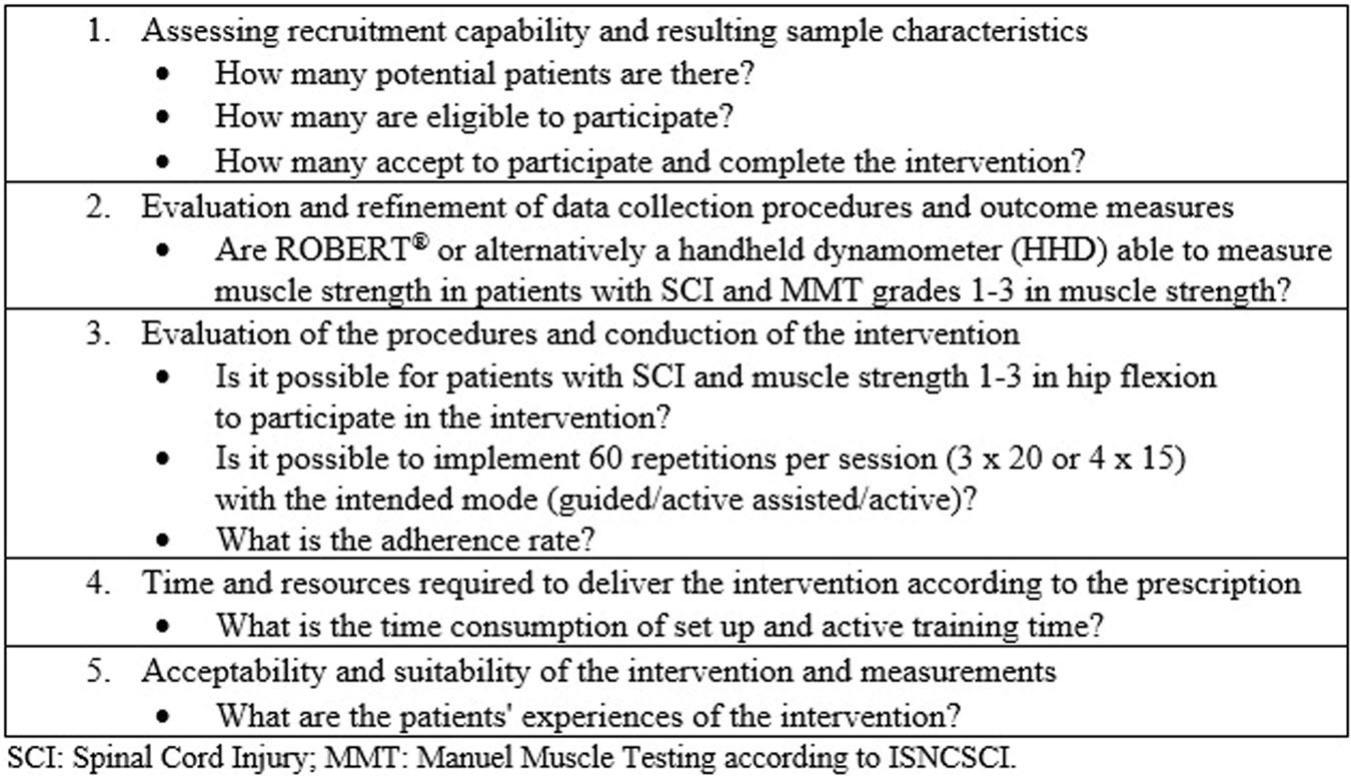
The parameters and research questions of the study.

This feasibility study was conducted at the SCI Centre of Western Denmark (SCICWD) preliminary to a future pilot RCT (NCT05558254). Inspired by Orsmond and Cohn [12], the parameters and research questions of the study are outlined in Fig. 1.

### Participants

Participants were recruited from consecutive admissions to SCICWD from November 2021 to June 2022. Potential participants were identified by the physiotherapists when they conducted the International Standards for Neurological Classification of Spinal Cord Injury (ISNCSCI 2019 revision [13]) evaluation. Inclusion criteria was: age ≥18 years, traumatic and non-traumatic SCI, <3 months from trauma/operation, and muscle strength grade 1–3 in both hip flexor muscles as determined by an MMT (conducted as part of the ISNCSCI). Strength in both hips was a consideration because one hip was trained and one hip was the control (the allocation of legs to trained and control was based on convenience because this was a feasibility study and we did not intend to present strength data on the two legs). Exclusion criteria was: previous cerebral injury/SCI, previous damage to the peripheral nervous system affecting the lower extremities, unstable fractures in the thorax or lower extremities, muscle strength of grade 0, 4, or 5 in the hip flexor muscles, weight >150 kg (due to limitations of ROBERT^®^). The project manager provided potential participants with information about the study and obtained written consent.

### Intervention

The intervention was pragmatically designed considering; i) what participants would tolerate and the likelihood of fatigue in very weak muscles and ii) what is realistic to implement into clinical physiotherapy practice. Twenty-five minutes three times a week was suggested to be the maximal time that could be assigned for muscle strength training for a single muscle. The intervention protocol consisted of 60 repetitions of hip flexion in supine for one leg conducted with assistance from the ROBERT^®^ three times a week for 4 weeks. This was provided in addition to usual practice. The other leg was not trained and acted as a control.

**Fig. 2:**
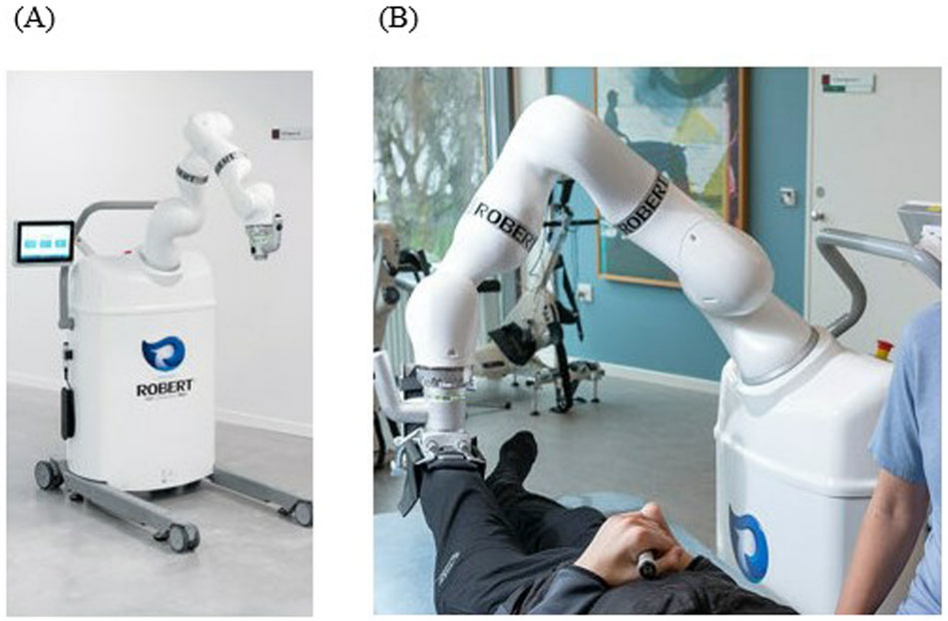
Illustration of the ROBERT^®^ and a typical set-up. **A** The ROBERT^®^. **B** Set-up where the ROBERT^®^ is attached to the lower extremity.

The first version of the ROBERT^®^ has been described elsewhere [14]. The upgraded model (Fig. 2) can perform exercises designed uniquely by the physiotherapist and can either operate in a guided or an active mode depending on the participant’s strength. A physiotherapist instructed in setting-up and operating the system is required to operate the ROBERT^®^.

The physiotherapist attached the ROBERT^®^ to the participant’s lower leg and guided the leg in a full ROM of hip flexion while ROBERT^®^ recorded the movement. For those with less than grade 3 strength (ie., unable to flex the hip against gravity), training was conducted in a guided mode in 3 sets of 20 repetitions, with 30 s of rest between each set. In this mode, the participant was encouraged to actively contribute to the guided movement as much as possible. For those with grade 3 (ie., able to flex the hip against gravity), the training was conducted in active mode in 4 sets of 15 repetitions, with 30 s of rest between each set. Resistance (from 1 to 9 - lowest to highest) was individually adjusted. When the participant was able to move through full ROM in one set resistance was increased. During practice, the participant received visual feedback on ROM and the number of repetitions.

### Usual training

Usual training consists of 45 min of individual physiotherapy 3–5 times a week. The sessions were individually adapted and consisted of exercise therapy, functional training, assistive devices, electrical stimulation, hydrotherapy, and treadmill training. The control leg did not receive any specific training that was not provided to the experimental leg as part of usual care.

## Data collection and analysis

### Quantitative data

All first-time admitted patients at SCICWD were registered and screened to assess recruitment capability. Once recruited, participants were assessed at baseline and after 4 weeks of training. Strength measures were taken of both hip flexor muscles (see results for details). Descriptive data of the dose and progression/deterioration of the training as well as data on the number of training sessions completed as intended and the time spent in ROBERT^®^ were recorded.

### Qualitative data

Interviews were conducted following a semi-structured interview guide to explore the participants’ experiences and perspectives of the acceptability and suitability of the intervention. The first author, who also was the provider of the intervention, conducted the interviews, transcribed them verbatim and analysed the interviews together with one of the co-authors using reflexive thematic analysis inspired by Braun and Clarke [15].

## Results

### Quantitative results

#### Recruitment capability and resulting sample characteristics

**Fig. 3:**
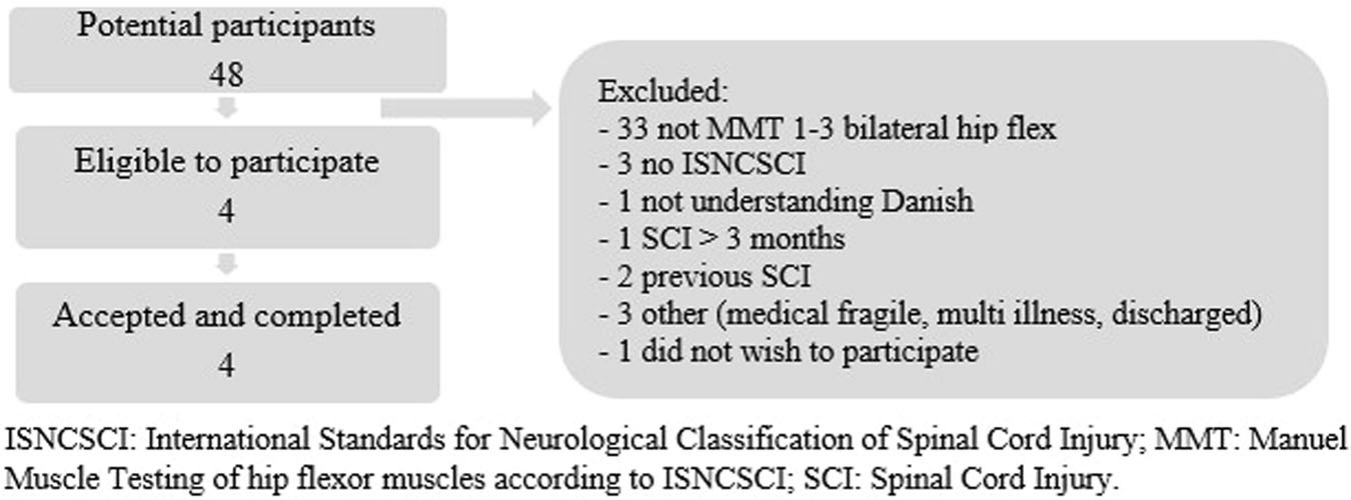
Flowchart recruitment from November 2021–June 2022.

We proposed to include four to eight participants in this feasibility study. From November 2021 until June 2022, 48 patients were registered as first-time admission to SCICWD. The flowchart of recruitment and completion is shown in Fig. 3. Due to the exclusion criteria, we excluded 2 patients with previous SCI, however, due to recruitment challenges, we decided to accept one participant with a previous SCI as we were primarily interested in the feasibility of administering the intervention. Four participants were recruited and all of them completed the study.

Characteristics of the four participants are shown in Table 1.

**Table 1:** Baseline demographic characteristics (*N* = 4).

Participant	Sex	Age	Aetiology	Time since injury (days)	Neurological level	AIS grade	MMT right hip flex	MMT left hip flex
ID 1	Male	86	Traumatic	31	C6	D	2	2
ID 2	Female	79	Non-traumatic	9	T10	C	2	1
ID 3	Male	64	Non-traumatic	71	C4	D	3	3
ID 4	Female	75	Non-traumatic	33	C2	D	2	3

*Time since injury* days from operation or trauma to the day for assessment with the International Standards for Neurological Classification of Spinal Cord Injury (ISNCSCI).

*AIS grade* American Spinal Injury Association Impairment Scale grade, *MMT* Manuel Muscle Testing of hip flexion according to ISNCSCI.

#### Evaluation and refinement of data collection procedures and outcome measures

**Fig. 4:**
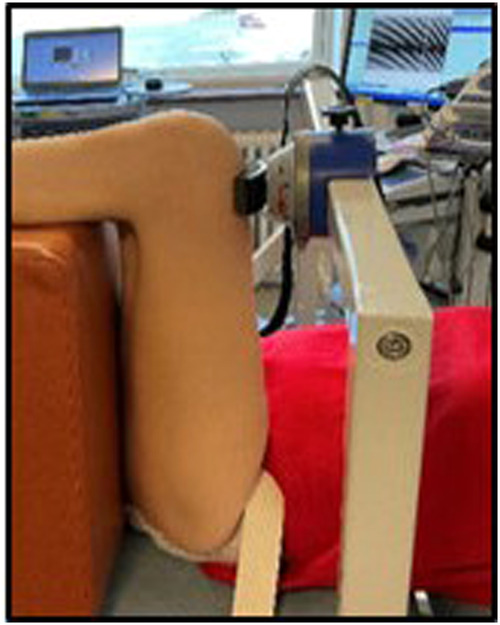
Test setup with the fixation of the dynamometer.

We looked at the feasibility of using the inbuilt force transducer of the ROBERT^®^ to measure muscle strength in the hip flexor muscles. However, this was not possible because it required the participant to perform 5–10° of active hip flexion. Those with grade 1 and 2 strength were not capable of doing this. Instead, we used a dynamometer MicroFET2, (Hoggan Scientific, Utah, USA) which has proven reliability and validity. The test protocol was standardised according to the literature [16, 17]. To increase reliability, we chose to fix the dynamometer to a frame to avoid the need for an assessor to hold it (Fig. 4).

The participant was positioned supine on the bed with the hip supported in 90° flexion. A strap was placed around the pelvis to maintain the contralateral hip in a neutral position. The dynamometer was located just proximal to the femoral condyles. Strength was measured using an isometric “make” test. The participant was instructed to maximally contract for 5 s followed by 30 s of rest. This was repeated three times. If the third time was highest the participants repeated the test once or twice more. The MVC in newton (N) was the primary outcome but all trials were recorded to describe the variation - see Table 2A, B.

**Table 2:** Muscle test (*N*) according to the protocol of 3–5 trials per participant.

Muscle test (N)	1. trial	2. trial	3. trial	4. trial	5. trial	Mean (N)	SD	CV
A) Muscle test (N) of the leg trained by ROBERT^®^								
ID 1 Baseline	75.6	86.7^a^	83.6			82.0	5.7	7.0
ID 1 4 weeks	98.7	120.5^a^	92.5			103.9	14.7	14.2
ID 2 Baseline^b^	-	-	-			-	-	-
ID 2 4 weeks	26.2	31.6^a^	30.2			29.3	2.8	9.6
ID 3 Baseline	93.4	94.3	96.1	108.5^a^	84.1	95.3	8.7	9.2
ID 3 4 weeks	149.9	157.5^a^	140.1			149.2	8.6	5.9
ID 4 Baseline	35.6	39.6	44.9	54.7^a^	54.3	45.8	8.6	18.7
ID 4 4 weeks	68.5	82.3^a^	77.0			75.9	7.0	9.2
B) Muscle test (N) of the control leg								
ID 1 Baseline	81.8^a^	55.2	60.5			65.8	14.1	21.4
ID 1 4 weeks	66.5	65.4	70.7^a^	62.5		66.3	3.4	5.1
ID 2 Baseline^b^	-	-	-			-	-	-
ID 2 4 weeks	148.1	153.5^a^	150.8			150.8	2.7	1.8
ID 3 Baseline	130.3^a^	116.5	124.5			123.8	6.9	5.6
ID 3 4 weeks	154.3^a^	128.5	141.9			141.6	12.9	9.1
ID 4 Baseline	56.5	64.1^a^	51.2			57.3	6.5	11.3
ID 4 4 weeks	49.4	64.9	81.8	82.3^a^	65.8	68.8	13.7	19.9

*SD* standard deviation, *CV* coefficient of variation (%).

^a^*MVC* maximal voluntary contraction,

^b^Missing baseline measure.

Seven out of eight tests were successful. One test failed because of the battery. The Coefficient of Variation (CV) in the individual’s measure of muscle strength (*N*) varied from 1.8% to 21.4%.

#### Evaluation of the procedures and conduction of the intervention

Three participants started in the guided mode. To explore the transition from guided mode to assisted active mode the two participants with grade 2 strength tried out the assisted active mode. The ROBERT^®^ allowed the participant to complete as much of the movement as possible before assisting. However, for ROBERT^®^ to complete the ROM, the participant needed to be able to stabilise the leg in a steady position (ie., stop the hip from extending, rotating or abducting). In addition, the participant had to initiate the movement and complete at least 30° of hip flexion before ROBERT^®^ offered assistance. The participants were not always able to do this and therefore the assistance was not consistently activated. Hence, they had to continue in the guided mode. One participant trained in the active mode. The resistance could not be increased because the participant could not complete full ROM for all repetitions.

The protocol dictated 60 repetitions per session. One participant completed all as intended. Another performed an additional 20 repetitions per session to see if she could complete this but reported being unable to actively contribute to the last set. Two had one session each where they ended the training before completing the 60 repetitions: one because of exhaustion, and one because of general discomfort not related to the training. No adverse events occurred during or after any sessions.

A total of 44 out of the intended 48 sessions were completed (Table 3). One session was cancelled due to a defect in the ROBERT^®^ and three were cancelled by the participants (reasons: 2 infections, 1 examination at another hospital).

**Table 3:** Exercise data including time required to set-up and exercise (minimum and maximal, and change in exercise duration (end-start)).

ID no.	Exercise mode	Sessions completed	Min. set-up time (min.:s.)	Max. set-up time (min.:s.)	Min. exercise time (min.:s.)	Max. exercise time (min.:s.)	Exercise time week 1 (min.:s.)	Exercise time week 4 (min.:s.)	Change (end- start) (min.:s.)
ID 1	Guided	11	4:49	12:12	10.28	14:36	14:27	11:51	−2:36
ID 2	Guided	12	4:47	11:00	12:00	16:28	13:38	15:09	1:31
ID 3	Active	11	3:50	5:42	7:35	16:09	11:39	7:42	−3:57
ID 4	Guided	10	7:59	9:01	11:23	15:57	15:57	11:28	−4:29

#### Time and resources required to deliver the intervention according to the protocol

Timed to set-up varied from 3 min. 50 s. to 12 min. 12 s. depending on how much assistance the participant required to move from the wheelchair to the bed. Once the ROBERT^®^ failed during set-up and needed to be restarted. The shortest active exercise time was 7 min 35 s and the longest was 16 min 28 s (Table 3). Exercises were completed faster in the active versus the guided mode. There was a tendency towards the participants completing the 60 repetitions faster in the last session compared to the first (Table 3). All sessions were completed within the allocated 25 min.

### Qualitative findings

#### Acceptability and suitability of the intervention

Findings from the semi-structured interviews were collapsed into two themes:

*I) “ROBERT*^®^ *as a good supplement to - but not a substitute for the physiotherapist”*

In general, the participants were positive towards having technology as part of their rehabilitation. All four participants stated that it had been a good experience to exercise with the ROBERT^®^. One stated that he was challenged more to his limits by the ROBERT^®^ than by his physiotherapist. The participants felt that specific exercises with the ROBERT^®^ were a good supplement to physiotherapy but that it could not replace a physiotherapist. Three of the participants perceived an increase in the strength in the intervention leg during the 4 weeks of training, but they could not be more specific about any functions that the exercise had improved. One did not perceive any change in strength. None of the participants had any adverse events.

*II) The “ROBERT*^®^ *motivates to exercise when training is individually planned and adjusted”*

The participants were very motivated to exercise. They did not find it tedious to repeat 60 hip flexions three times per week for four weeks. The three participants who conducted their exercises in the guided mode felt that the exercises were either of an appropriate difficulty or too easy. The one participant exercising in the active mode experienced the training as hard but suitable. All participants expressed a wish for more training and felt that the ROBERT^®^ provided good extra exercise. One participant’s foot pulled out of the boot while exercising, but apart from that, there were no other issues. Two participants emphasised the need to be encouraged during the exercises and that it was good that the exercises were adjusted by the physiotherapist. When asked if they could imagine being on their own when exercising with in the ROBERT^®^, two of the participants felt that it would be doable if they had the stop/start control and there was supervision by someone in the room.

## Discussion

This study aimed to determine the feasibility of conducting a definitive trial to investigate the effectiveness of the ROBERT^®^ for increasing strength in the very weak muscles following SCI. Specifically, we looked at the suitability and acceptability of the ROBERT^®^ for increasing strength in the hip flexor muscles. Our main findings were that the ROBERT^®^ was acceptable and suitable. These findings were based on adherence to the protocol and completion rate as well as from the participants’ perspectives. However, the recruitment was slow.

The participants were able to adhere to the training protocol of 60 hip flexion three times a week for four weeks. The adherence rate was high (92%). Only four sessions were cancelled. There were no adverse events. Considering the participants’ positive evaluation of the ROBERT^®^, the study showed that the intervention was feasible and acceptable. Based on the participants’ statements that exercising in the guided mode might not have been hard enough there needs to be a focus on the possibility of transferring the exercise from guided to active mode. This could be beneficial to explore further in a future study.

One physiotherapist was required to set-up and then supervise each session. The active exercise time varied from 7½ to 16½ min. for the 60 repetitions. If the intervention is proven to be effective, this seems an acceptable amount of time to devote to this type of training, however it may not be acceptable if there are many muscles that require the same amount of training, and they all need to be training one by one. Importantly, in the guided mode participants need to focus on actively contributing to the movement and not letting the ROBERT^®^ passively move the leg. In this study, the physiotherapist continuously motivated the participant to be as active as possible. These factors must be considered in the design of a definitive trial.

The recruitment rate was 8% of all first-time admissions to SCICWD over a period of 7 months. A randomised controlled trial investigating robotics as interventions for SCI in a similar setting but for participants with a high level of function (able to stand with assistance) showed a recruitment capability of 27% [18]. We could have increased our recruitment capacity if the design did not require bilateral hip flexor muscle weakness to enable comparison of the trained leg with the untrained leg. 69% of those screened were excluded because they were too strong. The use of this within participant type of design increases precision of treatment effects but also makes it more difficult to recruit. Future studies will need to weight up the pros and cons of these two competing factors. Recruitment would have been quicker if we did not need to restrict to very weak muscles however, the ROBERT^®^ is designed for these muscles. Recruitment will therefore pose a big challenge as described by others [19] and will require a multi-centre trial.

The dynamometer and test setup made it possible to measure strength (i.e. MVC) in the very weak hip flexor muscles. One criticism of using a handheld dynamometer (HHD) is the risk of the examiner providing an inconsistent counterforce and not holding the HHD perpendicular to the limb [17, 20, 21]. Both issues were eliminated in this study by the fixation of the dynamometer. There was variation in the individual MVC during the 3–5 trials per session, but all reached their maximum at the latest by the 4th trial. The CV in the individual’s measure of muscle strength (N) varied from 1.8% to 21.4%. Protocols for the HHD test often state one practice trial and two to three test trials [16, 17, 20]. Three of the 14 (21.4%) single-leg tests reached the MVC in the 4th trial. Participants having SCI might need more time to reach MVC. To ensure that they reach their MVC, it is probably prudent to allow five trials.

The participants perceived the ROBERT^®^ intervention as a good supplement to their usual rehabilitation and found it feasible and acceptable. We now plan to conduct a pilot trial to determine the likely sample size needed for a definitive multicentre trial.

## Data Availability

The dataset is available from the corresponding author upon reasonable request.
